# Comparison of the Performance of 6 Prognostic Signatures for Estrogen Receptor–Positive Breast Cancer: A Secondary Analysis of a Randomized Clinical Trial

**DOI:** 10.1001/jamaoncol.2017.5524

**Published:** 2018-02-15

**Authors:** Ivana Sestak, Richard Buus, Jack Cuzick, Peter Dubsky, Ralf Kronenwett, Carsten Denkert, Sean Ferree, Dennis Sgroi, Catherine Schnabel, Frederick L. Baehner, Elizabeth Mallon, Mitch Dowsett

**Affiliations:** 1 Centre for Cancer Prevention, Wolfson Institute of Preventive Medicine, Queen Mary University of London, London, England; 2 Ralph Lauren Centre for Breast Cancer Research, Royal Marsden, London, England; 3 Breast Cancer Now Research Centre, Institute of Cancer Research, London, England; 4 Department of Surgery and Comprehensive Cancer, Medical University of Vienna, Austria; 5 Breast Centre, Klinik St Anna, Luzern, Switzerland; 6 Sividon Diagnostics, GmbH, Köln, Germany; 7 Charité Universitätsmedizin and German Cancer Consortium, Berlin, Germany; 8 NanoString Technologies, Seattle, Washington; 9 Cancer Center, Massachusetts General Hospital, Boston, Massachusetts; 10 Biotheranostics, San Diego, California; 11 GenomicHealth, Redwood City, California; 12 School of Medicine, Dentistry, and Nursing, University of Glasgow, Glasgow, Scotland

## Abstract

**Importance:**

Multiple molecular signatures are available for managing estrogen receptor (ER)–positive breast cancer but with little direct comparative information to guide the patient’s choice.

**Objective:**

To conduct a within-patient comparison of the prognostic value of 6 multigene signatures in women with early ER-positive breast cancer who received endocrine therapy for 5 years.

**Design, Setting, and Participants:**

This retrospective biomarker analysis included 774 postmenopausal women with ER-positive *ERBB2* (formerly *HER2*)–negative breast cancer. This analysis was performed as a preplanned secondary study of data from the Anastrozole or Tamoxifen Alone or Combined randomized clinical trial comparing 5-year treatment with anastrozole vs tamoxifen with 10-year follow-up data. The signatures included the Oncotype Dx recurrence score, PAM50-based Prosigna risk of recurrence (ROR), Breast Cancer Index (BCI), EndoPredict (EPclin), Clinical Treatment Score, and 4-marker immunohistochemical score. Data were collected from January 2009, through April 2015.

**Main Outcomes and Measures:**

The primary objective was to compare the prognostic value of these signatures in addition to the Clinical Treatment Score (nodal status, tumor size, grade, age, and endocrine treatment) for distant recurrence for 0 to 10 years and 5 to 10 years after diagnosis. Likelihood ratio (LR) statistics were used with the χ^2^ test and C indexes to assess the prognostic value of each signature.

**Results:**

In this study of 774 postmenopausal women with ER-positive, *ERBB2*-negative disease (mean [SD] age, 64.1 [8.1] years), 591 (mean [SD] age, 63.4 [7.9] years) had node-negative disease. The signatures providing the most prognostic information were the ROR (hazard ratio [HR], 2.56; 95% CI, 1.96-3.35), followed by the BCI (HR, 2.46; 95% CI, 1.88-3.23) and EPclin (HR, 2.14; 95% CI, 1.71-2.68). Each provided significantly more information than the Clinical Treatment Score (HR, 1.99; 95% CI, 1.58-2.50), the recurrence score (HR, 1.69; 95% CI, 1.40-2.03), and the 4-marker immunohistochemical score (HR, 1.95; 95% CI, 1.55-2.45). Substantially less information was provided by all 6 molecular tests for the 183 patients with 1 to 3 positive nodes, but the BCI (ΔLR χ^2^ = 9.2) and EPclin (ΔLR χ^2^ = 7.4) provided more additional prognostic information than the other signatures.

**Conclusions and Relevance:**

For women with node-negative disease, the ROR, BCI, and EPclin were significantly more prognostic for overall and late distant recurrence. For women with 1 to 3 positive nodes, limited independent information was available from any test. These data might help oncologists and patients to choose the most appropriate test when considering chemotherapy use and/or extended endocrine therapy.

**Trial Registration:**

isrctn.com Identifier: ISRCTN18233230

## Key Points

**Question:**

What is the comparative performance of prognostic multigene signatures for estimation and risk stratification of overall and late distant recurrence in estrogen receptor–positive *ERBB2*-negative breast cancer?

**Findings:**

In this biomarker analysis of data from a randomized clinical trial, a combination of multigene expression tests with clinical information was associated with improved prognostic value for distant recurrences and risk stratification specifically in women with node-positive disease. Differences in the prognostic value for late distant recurrence were observed.

**Meaning:**

The combination of clinical and molecular information may enhance the prognostic value for distant recurrence and risk stratification in estrogen receptor–positive, *ERBB2*-negative breast cancer, particularly for women with node-positive disease.

## Teaser

This secondary analysis of a randomized clinical trial evaluates the prognostic value of 6 multigene signatures in women with early estrogen receptor–positive breast cancer who have received 5 years of endocrine therapy.

## Introduction

Almost all women with estrogen receptor (ER)–positive primary breast cancer are offered adjuvant endocrine therapy, and a highly relevant clinical question is who remains at high risk for distant recurrence despite completion of primary adjuvant therapy. Multigene expression profiles have significantly increased our ability to estimate distant recurrence in ER-positive breast cancer after surgery and endocrine treatment.^1^ These signatures are used in combination with different clinical characteristics to aid the selection of patients for whom chemotherapy may be appropriate based on prognosis. Several of these signatures are commercially available, including the Oncotype Dx recurrence score (RS) (Genomic Health), PAM50-based Prosigna risk of recurrence (ROR) (NanoString), Breast Cancer Index (BCI) (bioTheranostics), EndoPredict (EPclin) (Myriad Genetics), and MammaPrint Netherland Kanker Institute 70-gene signature (Agendia BV), are endorsed by several guidelines^2^ and are routinely used by clinicians.

The Translational Study of Anastrozole or Tamoxifen Alone or Combined (TransATAC) cohort was previously used to develop 2 prognostic algorithms, the Clinical Treatment Score (CTS), which includes clinicopathologic information, and the 4-marker immunohistochemical score (IHC4), which combines prognostic information of 4 widely used IHC markers.^6^ The following 4 gene expression–based signatures were also evaluated in the TransATAC cohort: the RS,^6^ ROR,^7^ BCI,^8^ and EPclin.^9^ The RS and BCI include only molecular information in their signatures, whereas the ROR (tumor size) and EPclin (tumor size and number of positive nodes) integrate clinical information. All these signatures significantly estimated the risk of distant recurrence, particularly in women with node-negative disease, but with a varying amount of prognostic information for late distant recurrence (5-10 years). An important area of research remains to accurately estimate the risk of late distant recurrence in women with ER-positive disease, because more than 50% of recurrences occur after 5 years of endocrine treatment. Gene expression–based signatures should show an improvement in prognostication when compared with standard clinical measures.^8^

To our knowledge, no direct and comprehensive comparison of multigene signatures has been performed in the same patient population with long-term follow-up data. We compared the prognostic performance of 6 signatures for distant recurrence in the 10-year period after diagnosis to assess the potential value of adding chemotherapy vs endocrine therapy alone and for late distant recurrence in years 5 to 10 to investigate the potential value of extended adjuvant endocrine therapy. Furthermore, the comparison was performed separately for women with node-negative disease and those with 1 to 3 positive nodes because the most significant prognostic clinical indicator for early-stage breast cancer is the presence or absence of lymph node involvement.

## Methods

### Study Design and Patients

In this preplanned comparative analysis, tumor blocks from the TransATAC study were used from patients with hormone receptor–positive early-stage breast cancer treated with 5 years of tamoxifen or anastrozole in the ATAC randomized clinical trial.^12^ Microdissection of the tumors and RNA extraction were performed by Genomic Health, Inc, and residual RNA was provided to collaborators for RNA expression profiling. Data were collected from January 2009, through April 2015. Women were excluded from the analysis if they received chemotherapy, did not have ER-positive disease, received the combination treatment (ie, anastrozole plus tamoxifen), or had 4 or more positive lymph nodes. This study was approved by the South-East London Research Ethics Committee. All patients provided written informed consent for their tissue to be used in translational research.

### Procedures

The CTS and IHC4 were developed in the TransATAC study and have been described in detail previously.^6^ In brief, the CTS contains information on nodal status, grade, tumor size, age, and treatment (tamoxifen vs anastrozole). The IHC4 combines 4 commonly used IHC markers, including ER, progesterone receptor, Ki67, and *ERBB2* (formerly known as *HER2* [RefSeq NM_001005862.2]). The commercial signatures are based on RNA expression profiling and were performed according to specifications by 4 of us (R.K., S.F., C.S., and F.L.B.) who were blinded to clinical outcome data. The RS^13^ is a 21-gene signature that was developed for patients with ER-positive, node-negative breast cancer. The RS risk groups were determined for patients with node-negative cancer as previously described,^13^ using predefined cutoffs of less than 18, 18 to 31, and greater than 31 to determine low-, intermediate-, and high-risk groups, respectively. The RS-pathology-clinical (RSPC) score was calculated using the website tool for patients with node-negative breast cancer.^14^ The BCI^15^ combines the 2-gene *HOXB13*:*IL17BR* ratio with the molecular grade index consisting of 5 proliferation genes in a linear model and was developed in postmenopausal patients with ER-positive, lymph node–negative breast cancer.^8^ Cutoff points for the BCI were determined in a population with node-negative disease (low risk <5.0825; high risk >6.5025).^17^ The ROR^7^ incorporates 46 genes and was developed in premenopausal and postmenopausal women treated without any adjuvant systemic therapy and includes information on tumor size. The TransATAC cohort was used to determine the cutoff points of the ROR for risk stratification in patients with node-negative and node-positive disease separately. They correspond approximately to a point estimate of as much as a 10% distant recurrence rate for low risk and more than a 20% rate for high risk after 10 years of follow-up.^18^ The EPclin was developed in premenopausal and postmenopausal tamoxifen-treated patients with ER-positive, *ERBB2*-negative breast cancer. It incorporates the expression of 12 genes plus information on tumor size and nodal status.^19^ A predefined cutoff point (3.3, based on methods described by Filipits et al^19^) was used for risk stratification, which corresponds to a 10% distant recurrence risk at 10 years.

### Statistical Analysis

The primary end point was the time to distant recurrence. Distant recurrence was defined as metastatic disease, excluding contralateral disease, and locoregional and ipsilateral recurrences. Death before distant recurrence was treated as a censoring event. We defined the following 2 primary analysis populations: patients with ER-positive, *ERBB2*-negative, node-negative breast cancer, and patients with ER-positive, *ERBB2*-negative breast cancer with 1 to 3 positive lymph nodes. The primary objective was the comparison of prognostic signatures in patients with node-negative and node-positive disease separately for overall (0-10 years) and late (5-10 years) follow-up periods.

We assessed overall distant recurrence in the first 10 years after diagnosis (n = 774) and late distant recurrence within the subset of patients who remained free of distant recurrence for the first 5 years after diagnosis (n = 689). Partial likelihood ratio (LR) tests based on Cox proportional hazards regression models were used to test the prognostic information of all signatures. The amount of prognostic information provided by each signature alone was assessed by C indexes. Furthermore, partial LR χ^2^ values with a 2-sided 5% significance level (LR χ^2^ = 3.84) are also presented. The improvement in distant recurrence prognostication of each signature compared with clinical and pathologic variables (CTS) was quantified by the increase in the LR χ^2^ value (ΔLR χ^2^; 2-sided 5% significance level). Predefined cutoffs were used to determine risk stratification for the 4 commercially available signatures. Kaplan-Meier curves were used to estimate the mean risk of distant recurrence after 10 years of follow-up in predefined risk groups. To compare the prognostic performance of all signatures, continuous scores were normalized to have unit variance, and the hazard ratios (HRs) and associated 95% CIs were estimated from Cox proportional hazards regression models. All statistical analyses were 2-sided, and a *P* value of less than .05 was regarded as significant. All analyses were performed with Stata software (version 13.1; StataCorp).

## Results

A total of 774 postmenopausal women with ER-positive, *ERBB2*-negative disease for whom all signatures were available were included in this analysis (mean [SD] age, 64.1 [8.1] years) (eFigure 1 in the Supplement). Five hundred ninety-one women had node-negative disease, with a mean (SD) age of 63.4 (7.9) years and a mean (SD) tumor size of 17.6 (8.5) mm. A total of 58 distant recurrences (9.8%) were recorded for this population, with approximately half of distant recurrences (n = 34) occurring in the late follow-up period (eTable 1 in the Supplement). In contrast, women with 1 to 3 positive nodes (n = 183) were significantly older (mean [SD] age, 66.4 [8.3] years) and had significantly larger tumors (mean [SD] size, 24.2 [12.2] mm) than those with negative nodes (eTable 1 in the Supplement). Forty distant recurrences were recorded during 10 years of follow-up, with 21 of them occurring 5 years after the diagnosis. Results of the prognostic performance of all 6 signatures for the overall population (with node-negative and node-positive disease combined) and C indexes are shown in eTable 2 in the Supplement.

### Recurrence During Years 0 to 10

#### Population With Node-Negative Disease

All 6 signatures provided a statistically significant prognostic value for distant recurrence during years 0 to 10; all HRs and C indexes are shown in Table 1. The ROR (HR, 2.56; 95% CI, 1.96-3.35), BCI (HR, 2.46; 95% CI, 1.88-3.23), and EPclin (HR, 2.14; 95% CI, 1.71-2.68) proved to be statistically more prognostic than the other signatures in this patient population. The CTS (HR, 1.99; 95% CI, 1.58-2.50) and IHC4 (HR, 1.95; 95% CI, 1.55-2.45) provided similar amounts of prognostic information in this period (Table 1 and eFigure 2 in the Supplement). All signatures provided independent prognostic information beyond the CTS for women with node-negative disease; in particular, the BCI and ROR provided the most prognostic value (eFigure 2 in the Supplement).

**Table 1.** Univariate HRs and C Indexes for All Prognostic Signatures According to Nodal Status During Years 0 to 10

Gene Signature	Patient Group			
	Node-Negative Disease (n = 591)		Node-Positive Disease (n = 227)	
	HR (95% CI)^a^	C Index (95% CI)	HR (95% CI)^a^	C Index (95% CI)
CTS	1.99 (1.58-2.50)	0.721 (0.668-0.774)	1.63 (1.20-2.21)	0.640 (0.554-0.726)
IHC4	1.95 (1.55-2.45)	0.725 (0.665-0.785)	1.33 (0.99-1.78)	0.601 (0.511-0.690)
RS	1.69 (1.40-2.03)	0.667 (0.585-0.750)	1.39 (1.05-1.85)	0.603 (0.513-0.693)
BCI	2.46 (1.88-3.23)	0.762 (0.704-0.820)	1.67 (1.21-2.29)	0.652 (0.566-0.739)
ROR	2.56 (1.96-3.35)	0.764 (0.707-0.821)	1.58 (1.16-2.15)	0.636 (0.552-0.719)
EPclin	2.14 (1.71-2.68)	0.765 (0.716-0.814)	1.69 (1.29-2.22)	0.671 (0.590-0.752)

Abbreviations: BCI, Breast Cancer Index; CTS, Clinical Treatment Score; EPclin, EndoPredict clinical score; HR, hazard ratio; IHC4, 4-gene immunohistochemical score; ROR, risk of recurrence; RS, recurrence score.

^a^ All HRs indicate a change in 1 SD.

**Figure 1.**
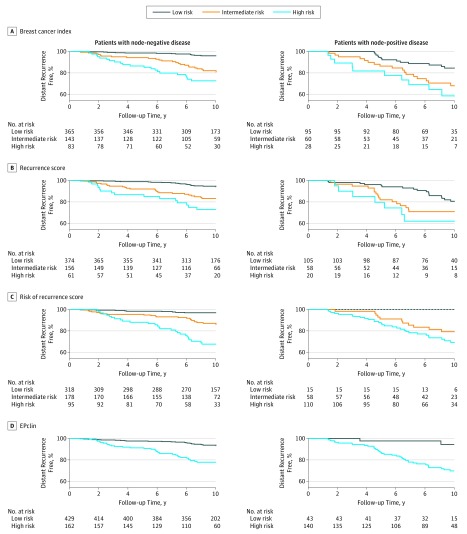
Kaplan-Meier Curves for Recurrence During Years 0 to 10 Includes 591 patients with node-negative and 183 patients with node-positive disease. Data are stratified by signature and nodal status. Gene signatures include the Oncotype Dx recurrence score (Genomic Health), PAM50-based Prosigna risk of recurrence score (NanoString), Breast Cancer Index (bioTheranostics), and EPclin (EndoPredict clinical score [Myriad Genetics]). Cutoffs for risk of recurrence score were trained separately in patients with node-negative and node-positive disease in the Translational Study of Anastrozole or Tamoxifen Alone or Combined cohort.

We determined 10-year distant recurrence risks for the 4 commercially available multigene signatures using predefined cutoffs (Figure 1 and Table 2). All 4 signatures identified a large proportion of women who were at low risk of developing a distant recurrence (<10%) after 10 years of follow-up. The EPclin signature has only 2 risk groups and categorized 429 women (72.6%) into the low-risk group, of whom 27 developed a distant recurrence (10-year distant recurrence risk, 6.6%; 95% CI, 4.5%-9.7%) (Figure 1 and Table 2). Only 61 patients (10.3%) were categorized into the high-risk group by the RS, and they had a 10-year distant recurrence risk of 27.2% (95% CI, 17.3%-41.2%). The EPclin, BCI, and ROR identified larger proportions of women as having a high risk with 10-year distant recurrence risks of 22.1% (95% CI, 16.2%-29.8%), 27.3% (95% CI, 18.7%-38.8%), and 32.4% (95% CI, 23.4%-43.8%), respectively (Figure 1 and Table 2). For 507 women, information for the RSPC score was also available, and the incorporation of clinical variables into the RS substantially improved the prognostic performance for distant recurrence compared with the molecular RS alone.

**Table 2.** Risk of Distant Recurrence Among Women With Node-Negative and Node-Positive Disease by Gene Signature

Gene Signature	Node-Negative Disease		Node-Positive Disease	
	No. (%)	10-y Risk, % (95% CI)	No. (%)	10-y Risk, % (95% CI)
**Years 0-10**				
BCI				
Low	365 (61.8)	3.9 (2.3-6.7)	95 (51.9)	15.5 (9.3-25.3)
Intermediate	143 (24.2)	19.3 (13.3-27.6)	60 (32.8)	32.0 (21.1-46.6)
High	83 (14.0)	27.3 (18-7-38.8)	28 (15.3)	41.4 (24.3-64.1)
RS				
Low	374 (63.3)	5.9 (3.8-9.1)	105 (57.4)	19.4 (12.5-29.5)
Intermediate	156 (26.4)	16.7 (11.5-24.0)	58 (31.7)	29.1 (18.9-43.1)
High	61 (10.3)	27.2 (17.3-41.2)	20 (10.9)	38.0 (20.0-64.1)
ROR				
Low	318 (53.8)	3.0 (1.6-5.8)	15 (8.2)	0
Intermediate	178 (30.1)	14.1 (9.4-20.8)	58 (31.7)	20.7 (12.0-34.4)
High	95 (16.1)	32.4 (23.4-43.8)	110 (60.1)	30.7 (22.2-41.3)
EPclin				
Low	429 (72.6)	6.6 (4.5-9.7)	43 (23.5)	5.6 (1.4-20.9)
High	162 (27.4)	22.1 (16.2-29.8)	140 (76.5)	30.3 (23.0-39.3)
**Years 5-10**^a^				
BCI				
Low	340 (63.6)	2.6 (1.3-5.0)	84 (54.6)	9.5 (8.3-23.9)
Intermediate	126 (23.6)	14.4 (9.0-22.6)	50 (32.5)	14.3 (8.3-23.9)
High	69 (12.8)	15.9 (8.9-27.6)	20 (13.0)	36.5 (20.4-59.6)
RS				
Low	351 (65.6)	4.8 (2.9-7.9)	94 (61.0)	17.9 (11.5-27.3)
Intermediate	134 (25.1)	9.6 (5.6-16.3)	45 (29.2)	19.5 (10.9-33.5)
High	50 (9.3)	16.1 (8.0-30.8)	15 (9.7)	27.5 (11.2-57.9)
ROR				
Low	292 (54.6)	1.4 (0.5-3.8)	15 (9.7)	0
Intermediate	165 (30.8)	10.0 (6.0-16.5)	51 (33.1)	13.0 (6.1-26.7)
High	78 (14.6)	23.2 (14.9-35.2)	88 (57.1)	25.0 (17.5-35.0)
EPclin				
Low	393 (73.5)	4.3 (2.6-7.1)	40 (26.0)	3.3 (0.5-21.4)
High	142 (26.5)	14.6 (9.6-22.0)	114 (74.0)	23.6 (17.0-32.1)

Abbreviations: BCI, Breast Cancer Index; EPclin, EndoPredict clinical score; HR, hazard ratio; IHC4, 4-gene 5 immunohistochemical score; ROR, risk of recurrence; RS, recurrence score.

^a^ Percentages have been rounded and may not total 100.

#### Population With 1 to 3 Positive Nodes

The CTS (HR, 1.63; 95 CI, 1.20-2.21), BCI (HR, 1.67; 95% CI, 1.21-2.29), ROR (HR, 1.58; 95% CI, 1.16-2.15), and EPclin (HR, 1.69; 95% CI, 1.29-2.22) provided significant prognostic information in this patient population (Table 1). The prognostic performance of all signatures, although significant, was weaker than for node-negative disease, as evidenced by the smaller HRs and C indexes in this patient group. The IHC4 did not provide prognostic value for distant recurrence. Apart from the IHC4, all signatures provided independent prognostic information, with the BCI (ΔLR χ^2^ = 9.2) and EPclin (ΔLR χ^2^ = 7.4) showing the largest improvements beyond the CTS (eFigure 2 in the Supplement).

Risk group stratification is shown in Figure 1 and Table 2. The ROR identified a small group of women (n = 15) as low risk, of whom none developed a distant recurrence at 10 years (Figure 1 and Table 2). The EPclin categorized 43 women (23.5%) into the low-risk group, with a 5.6% (95% CI, 1.4%-20.9%) risk of distant recurrence at 10 years. Both signatures identified most women as high risk with a mean 10-year distant recurrence risk of more than 30%. In contrast, a high proportion of women were categorized into the low-risk group with a high risk of distant recurrence at 10 years by the BCI (15.5%; 95% CI, 9.3%-25.3%) and the RS (19.4%; 95% CI, 12.5%-29.5%) (Figure 1 and Table 2).

### Recurrence During Years 5 to 10

#### Population With Node-Negative Disease

To assess the prognostic power of each signature for late distant recurrence, 535 women who were alive and without distant recurrence after 5 years of follow-up were included. The HRs and C indexes are shown in Table 3. The ROR (HR, 2.77; 95% CI, 1.93-3.96), BCI (HR, 2.30; 95% CI, 1.61-3.30), and EPclin (HR, 2.19; 95% CI, 1.62-2.97) provided significant prognostic value for late distant recurrence (Table 3 and eFigure 3 in the Supplement) and substantially more than the CTS alone (HR, 1.95; 95% CI, 1.43-2.65). The IHC4 and RS did not provide significant prognostic value for late distant recurrence when added to CTS (eFigure 3 in the Supplement). The BCI (ΔLR χ^2^ = 11.2), EPclin (ΔLR χ^2^ = 10.3), and, in particular, ROR (ΔLR χ^2^ = 18.4) provided significant independent prognostic information for late distant recurrence beyond the CTS (eFigure 3 in the Supplement). The RSPC score provided twice as much prognostic information for late distant recurrence compared with the RS alone in the univariate analysis but no prognostic value for late distant recurrence additional to CTS.

**Table 3.** Univariate HRs and C Indexes for All Prognostic Signatures According to Nodal Status During Years 5 to 10

Gene Signature	Patient Group			
	Node-Negative Disease (n = 535)		Node-Positive Disease (n = 154)	
	HR (95% CI)^a^	C Index (95% CI)	HR (95% CI)^a^	C Index (95% CI)
CTS	1.95 (1.43-2.65)	0.721 (0.654-0.788)	1.61 (1.05-2.47)	0.644 (0.534-0.753)
IHC4	1.59 (1.16-2.16)	0.660 (0.576-0.745)	1.20 (0.79-1.81)	0.579 (0.460-0.697)
RS	1.46 (1.09-1.96)	0.585 (0.467-0.702)	1.24 (0.81-1.90)	0.555 (0.418-0.693)
BCI	2.30 (1.61-3.30)	0.749 (0.668-0.830)	1.60 (1.04-2.47)	0.633 (0.514-0.751)
ROR	2.77 (1.93-3.96)	0.789 (0.724-0.854)	1.65 (1.08-2.51)	0.643 (0.528-0.758)
EPclin	2.19 (1.62-2.97)	0.768 (0.701-0.835)	1.87 (1.27-2.76)	0.697 (0.594-0.799)

Abbreviations: BCI, Breast Cancer Index; CTS, Clinical Treatment Score; EPclin, EndoPredict clinical score; HR, hazard ratio; IHC4, 4-gene immunohistochemical score; ROR, risk of recurrence; RS, recurrence score.

^a^ All HRs indicate a change in 1 SD.

**Figure 2.**
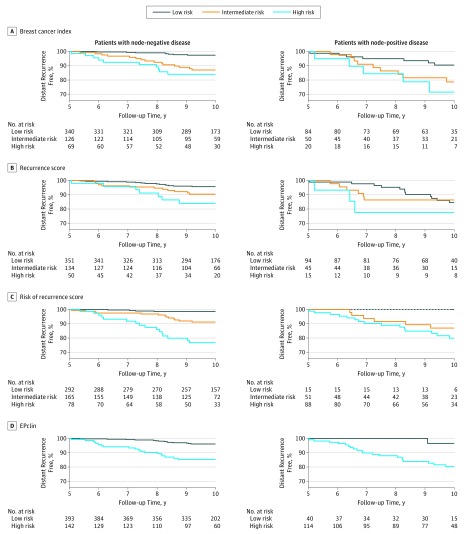
Kaplan-Meier Curves for Recurrence During Years 5 to 10 Includes 535 patients with node-negative and 154 with node-positive disease. Data are stratified by signature and nodal status. Gene signatures include the Oncotype Dx recurrence score (Genomic Health), PAM50-based Prosigna risk of recurrence score (NanoString), Breast Cancer Index (bioTheranostics), and EPclin (EndoPredict clinical score [Myriad Genetics]). Cutoffs for risk of recurrence score were trained separately in patients with node-negative and node-positive disease in the Translational Study of Anastrozole or Tamoxifen Alone or Combined cohort.

All 4 signatures categorized most of the women into the low-risk group, who had a low mean distant recurrence risk in years 5 to 10 of less than 5% (Figure 2 and Table 2). The EPclin categorized 142 patients (26.5%) into the high-risk group, which had the lowest 10-year distant recurrence risk of 14.6% (95% CI, 9.6%-22.0%). In contrast, the ROR identified 78 women (14.6%) as high risk, and they had the highest 10-year risk of distant recurrence of any test (23.2%; 95% CI, 14.9%-35.2%) (Figure 2 and Table 2).

#### Population With 1 to 3 Positive Nodes

We included 154 women who were alive without distant recurrence within the first 5 years of follow-up (Table 3 and eFigure 3 in the Supplement). The EPclin provided the most prognostic value for late distant recurrence (HR, 1.87; 95% CI, 1.27-2.76), followed by the ROR (HR, 1.65; 95% CI, 1.08-2.51) and BCI (HR, 1.60; 95% CI, 1.04-2.47) (Table 3). The IHC4 and RS did not provide prognostic information for late distant recurrence univariately or in addition to the CTS (eFigure 3 in the Supplement). The EPclin (ΔLR χ^2^ = 6.1) and BCI (ΔLR χ^2^ = 4.6) added significant but limited independent prognostic information to the CTS (eFigure 3 in the Supplement).

Good risk stratification in this patient group was observed for the BCI, ROR, and EPclin (Figure 2 and Table 2). The ROR categorized 15 women (9.7%) into the low-risk group, of whom none developed a late distant recurrence. The EPclin identified a larger proportion of women as low risk (40 [26.0%]), of whom only 1 developed a distant recurrence by year 10. No clear risk stratification was observed for the RS (Figure 2 and Table 2).

## Discussion

Multigene signatures have become increasingly important for the prognostic evaluation of ER-positive, *ERBB2*-negative breast cancer.^6^ Herein we compared the prognostic value of 6 signatures for distant recurrence in the TransATAC cohort. During years 0 to 10, all signatures provided significant prognostic information for women with node-negative disease in addition to clinical variables. For women with 1 to 3 positive nodes, the independent prognostic strength of the investigated signatures was weaker. Although fewer patients had node-positive than node-negative disease, the number of distant recurrences was similar and thus provided similar power. For late distant recurrence, the BCI, ROR, and EPclin provided independent prognostic information for women with node-negative disease and those with 1 to 3 positive nodes.

We previously published the results of the individual evaluations of the 4 commercial signatures and showed that all provide significant and similar prognostic information during the first 5 years after diagnosis.^6^ In this study, we revealed that the difference in prognostic performance between signatures during 10 years of follow-up may have been largely attributable to their differential ability to estimate distant recurrence from 5 to 10 years. Thus, the BCI,^8^ ROR,^23^ and EPclin^9^ may have molecular components in their signatures that are more specifically prognostic for late recurrence compared with the IHC4 and the RS. Of importance, combined genomic and clinical models showed enhanced prognostic performance particularly for patients with 1 to 3 positive lymph nodes and thus may be the preferred approach for the decision-making process for this patient group. Furthermore, the RSPC score provided significantly more prognostic value for distant recurrence in patients with node-negative disease than did the molecular RS alone.

In the adjuvant setting, the need for chemotherapy or extended endocrine therapy (for late recurrence) is an important clinical decision. We used predefined cutoffs to determine the 10-year distant recurrence risk for the commercial scores during years 0 to 10 (chemotherapy) and 5 to 10 (extended endocrine therapy). For node-negative disease, most of the women were categorized into the low-risk group by all 4 signatures, and women had a mean risk of less than 7%, for whom chemotherapy might not be indicated. The 2 signatures that contain clinical variables (ROR and EPclin) identified a sizeable group of women with 1 to 3 positive nodes who had a very low risk of distant recurrence at 10 years (mean risk, <6%), suggesting that chemotherapy would be of very limited benefit in these women.

None of the signatures were specifically developed to estimate late distant recurrences. However, the BCI, ROR, and EPclin demonstrated accurate prognostic value for these late events in our analysis. Wolmark and colleagues^24^ reported that the RS was significantly prognostic for late distant recurrence but only in patients with high *ESR1* levels. However, we did not observe an association between high *ESR1* levels and late distant recurrence with the RS in our data set. A few studies^25^ have investigated a series of extended endocrine therapy with aromatase inhibitors to address the question of the ideal length of extended treatment. The MA17.R trial^25^ showed that 10 years of letrozole therapy resulted in significantly higher rates of disease-free survival compared with placebo. In the National Surgical Adjuvant Breast and Bowel Project (NSABP-B42),^26^ extended adjuvant aromatase inhibition after sequential endocrine therapy (DATA),^28^ and Investigation on the Duration of Extended Adjuvant Letrozole (IDEAL)^27^ trials, no significant improvement in disease-free or overall survival with extended endocrine therapy was observed. These data raise the question of whether patients need to be specifically selected for extended endocrine therapy (ie, based on high risk for late distant recurrence or high likelihood of benefit from extended therapy).

### Strengths and Limitations

Strengths of our study include the mature clinical data with clinical outcome and long-term follow-up, well-characterized tissue samples, and data on 6 prognostic signatures for breast cancer. For all RNA analyses, the same extraction methods for RNA were used. For all commercial signatures, standardized quantitative methods and analyses were used, and all collaborators were blinded to clinical outcomes. Limitations include that our results are only applicable for chemotherapy-free and postmenopausal women. An unintended selection bias might have occurred because sample analyses might have only been possible where sufficient amounts of RNA were available, but all assays yielded reportable results. The IHC4, CTS, and in part the RSPC score were trained in the TransATAC cohort, and thus their performance may have been overestimated in this analysis. The risk group cutoffs of the ROR were defined in the TransATAC cohort for women with node-negative and node-positive disease separately, therefore optimizing the cutoffs to identify a low-risk group with less than 10% risk and a high-risk group with greater than 20% risk. Finally, our current analysis was not able to assess the ability of these signatures to predict the benefit from chemotherapy or extended endocrine therapy.

## Conclusions

The prognostic signatures evaluated provided significant information to help determine appropriate candidates consisting of patients with ER-positive, *ERBB2*-negative breast cancer, for whom chemotherapy and extended endocrine therapy might not be indicated. In patients with node-negative disease, all multigene signatures provided significant and clinically meaningful prognostic information beyond clinical factors. The combination of clinical and molecular information enhanced prognostic performance, particularly for women with node-positive disease. All signatures performed similarly during the first 5 years of follow-up, but we found differences during years 5 to 10, when these tests may be valuable for decision making with regard to extended endocrine treatment.

## Notes

Supplement. **eFigure 1.** CONSORT Diagram **eFigure 2.** Likelihood Ratio χ^2^ Values for Years 0-10 **eFigure 3.** Likelihood Ratio χ^2^ Values for Years 5-10 **eTable 1.** Baseline Characteristics According to Nodal Status **eTable 2.** C Indexes and Likelihood Ratio χ^2^ Values
