# Supplementary material for: Comparison of the Performance of 6 Prognostic Signatures for Estrogen Receptor–Positive Breast Cancer: A Secondary Analysis of a Randomized Clinical Trial
Source: JAMA Oncol. 2018 Feb 15;4(4):545–53. doi: 10.1001/jamaoncol.2017.5524 (PMC5885222; doi:10.1001/jamaoncol.2017.5524)
Supplement: Supplement. — eFigure 1. CONSORT Diagram eFigure 2. Likelihood Ratio χ2 Values for Years 0-10 eFigure 3. Likelihood Ratio χ2 Values for Years 5-10 eTable 1. Baseline Characteristics According to Nodal Status eTable 2. C Indexes and Likelihood Ratio χ2 Values [file jamaoncol-4-545-s001.pdf]

Supplementary Online Content

Sestak I, Buus R, Cuzick J, et al. Comparison of the performance of 6 prognostic signatures for estrogen receptor–positive breast cancer: a secondary analysis of a randomized clinical trial. *JAMA Oncol*. Published online February 15, 2017. doi:10.1001/jamaoncol.2017.5524

eFigure 1. CONSORT Diagram

eFigure 2. Likelihood Ratio χ^2 Values for Years 0-10

eFigure 3. Likelihood Ratio χ^2 Values for Years 5-10

eTable 1. Baseline Characteristics According to Nodal Status

eTable 2. C Indexes and Likelihood Ratio χ^2 Values

This supplementary material has been provided by the authors to give readers additional information about their work.

© 2018 Sestak et al. *JAMA Oncology.*

eFigure 1. CONSORT Diagram

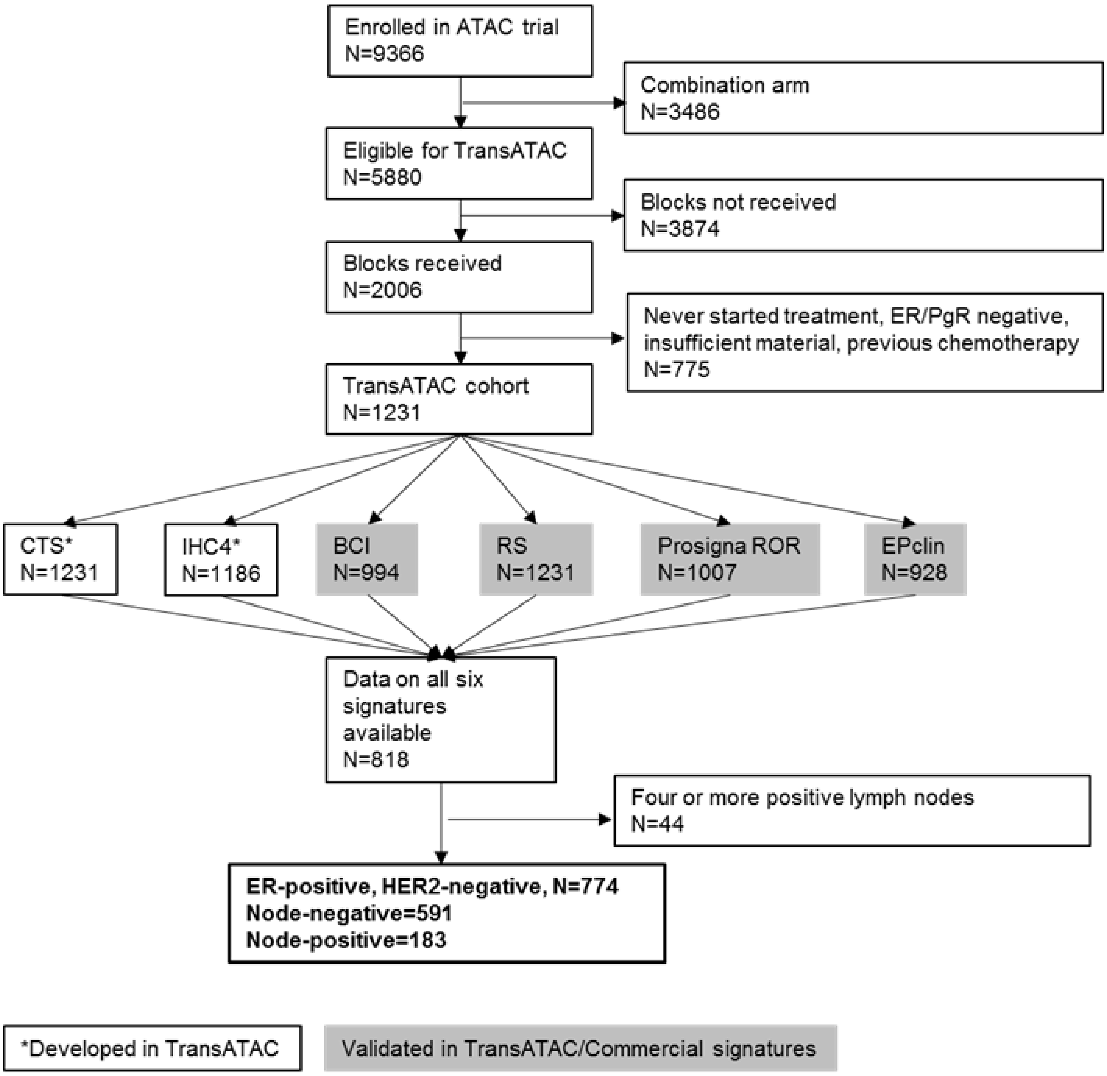

ATAC=Arimidex Tamoxifen Alone or in Combination, ER=Oestrogen Receptor, PgR=Progesterone Receptor, CTS=Clinical Treatment Score, IHC4=Immunohistochemical Score, BCI=Breast Cancer Index, RS=Recurrence Score, ROR=Risk of Recurrence Score, EPclin=EndoPredict clinical

© 2018 Sestak et al. *JAMA Oncology.*

eFigure 2. Likelihood Ratio χ^2 Values for Years 0-10

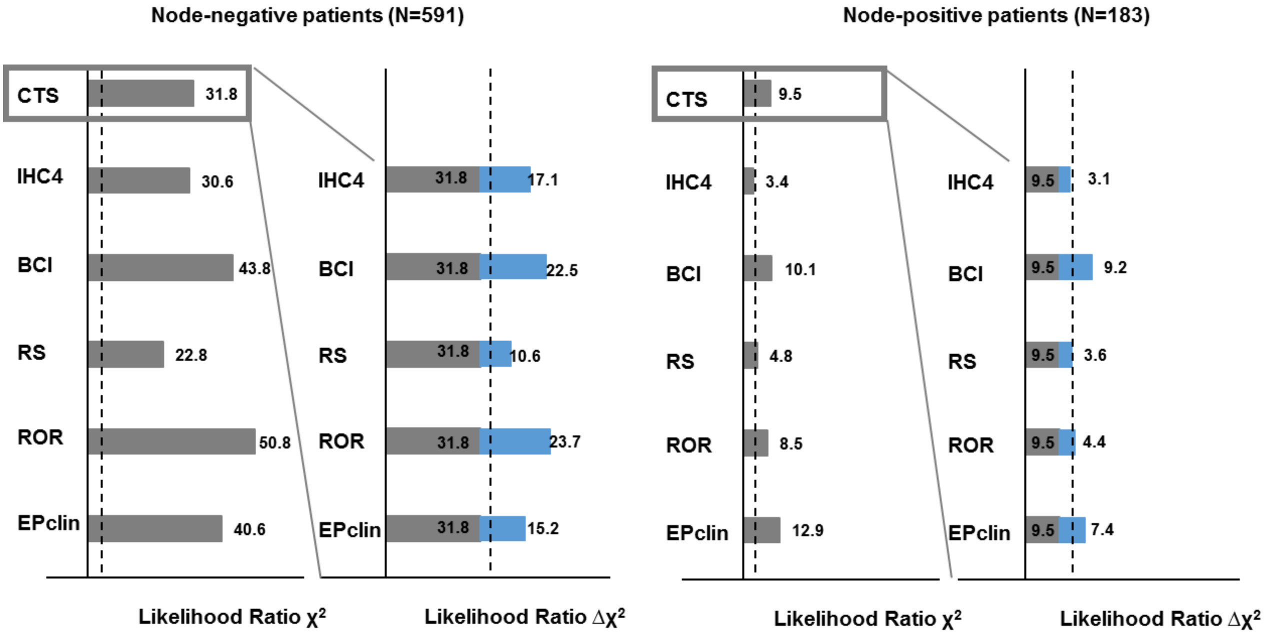

CTS=Clinical Treatment Score, IHC4=Immunohistochemical Score, BCI=Breast Cancer Index, RS=Recurrence Score, ROR=Risk of Recurrence Score,EPclin=EndoPredict clinical

Values are given for prognostic information of each signature on its own (LR-χ2) and when added to CTS (ΔLR-χ2) in years 0-10 for node-negative patients (left) and node-positive patients (right). Dotted line indicates significance level of 5% (LR-χ2=3.84).

© 2018 Sestak et al. *JAMA Oncology.*

eFigure 3. Likelihood Ratio χ^2 Values for Years 5-10

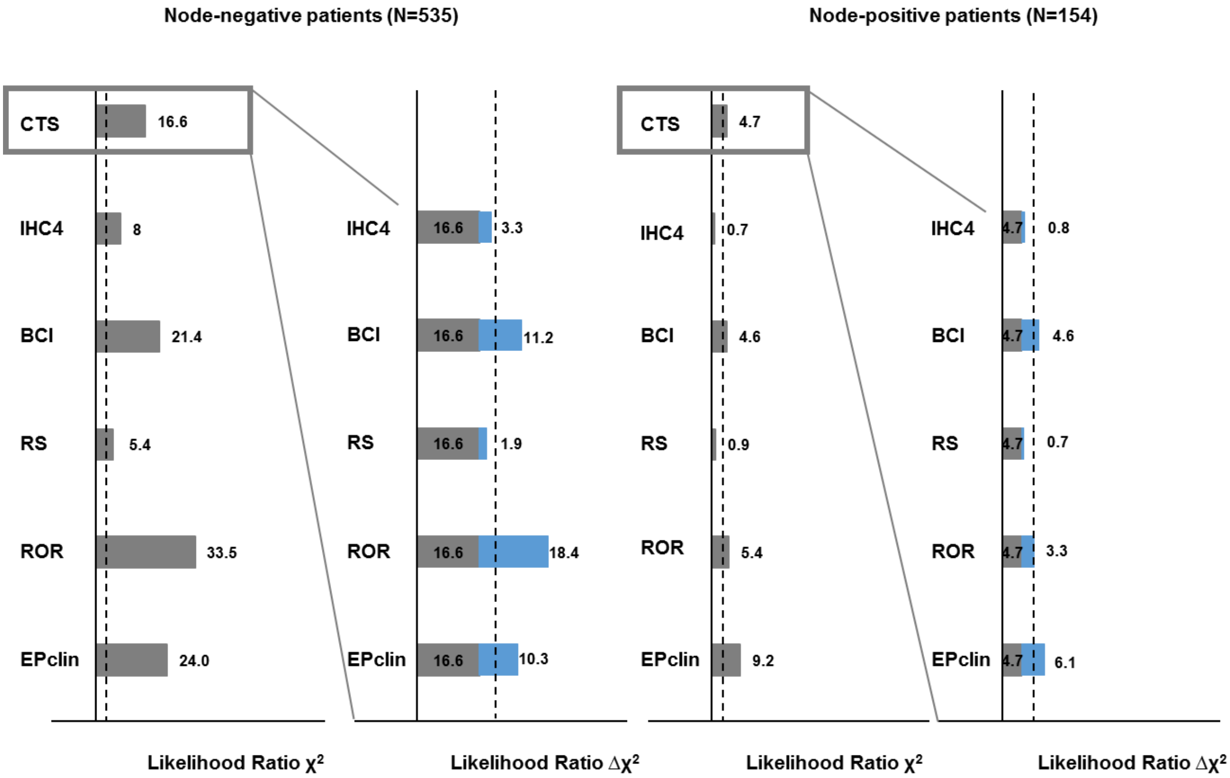

CTS=Clinical Treatment Score, IHC4=Immunohistochemical Score, BCI=Breast Cancer Index, RS=Recurrence Score, ROR=Risk of Recurrence Score,EPclin=EndoPredict clinical

Values are given for prognostic information of each signature on its own (LR-χ2) and when added to CTS (ΔLR-χ2) in years 5-10 for node-negative patients (left) and node-positive patients (right). Dotted line indicates significance level of 5% (LR-χ2=3.84).

© 2018 Sestak et al. *JAMA Oncology.*

eTable 1. Baseline Characteristics According to Nodal Status

	Node-negative (N=591)	Node-positive (N=183)
**Mean age, years (SD)**	63.4 (7.9)	66.4 (8.3)
**Mean BMI, kg/m^2 (SD)**	27.3 (4.9)	26.7 (4.7)
**Grade**		
1	23.2%	21.9%
2	59.7%	58.5%
3	17.1%	19.7%
**Mean tumour size, mm (SD)**	17.6 (8.5)	24.2 (12.2)
**Distant recurrence**		
**0-10 years**	58 (9.8%)	40 (21.9%)
**5-10 years**	34/535 (6.4%)	21/154 (13.6%)

SD=Standard Deviation, kg-kilogram, m=metre, mm=millimetre

© 2018 Sestak et al. *JAMA Oncology.*

eTable 2. C Indexes and Likelihood Ratio χ^2 Value

	C-index (95% CI)	LR-χ^2	∆LR-χ^2	Number of women in risk group (low/intermediate/high)	10-year DR risk (low/intermediate/high)
	**Years 0-10 (N=774)**				
CTS	0.726 (0.683-0.768)	55.3	-	-	-
IHC4	0.680 (0.630-0.731)	31.7	20.1	-	-
BCI	0.727 (0.680-0.744)	55.8	32.8	460/203/111	6.3%/23.0%/30.6%
RS	0.645 (0.584-0.706)	26.9	15.2	479/214/81	8.7%/20.1%/29.9%
ROR (no tumor size)	0.716 (0.668-0.763)	56.2	27.4	-	-
ROR	0.725 (0.679-0.772)	61.5	26.3	333/236/205	2.9%/15.7%/31.4%
EP	0.705 (0.658-0.752)	42.7	23.7	323/-/451	5.5%/-/20.1%
EPclin	0.753 (0.713-0.793)	69.3	24.4	472/-/302	6.5%/-/25.9%
	**Years 5-10 (N=689)**				
CTS	0.725 (0.671-0.779)	28.2	-	-	-
IHC4	0.634 (0.566-0.702)	8.3	4.5	-	-
BCI	0.717 (0.651-0.782)	27.4	16.5	424/176/89	3.9%/16.2%/18.6%
RS	0.574 (0.485-0.662)	6.3	3.0	445/179/65	6.9%/10.6%/17.6%
ROR (no tumor size)	0.729 (0.667-0.790)	35.1	19.0	-	-
ROR	0.745 (0.687-0.804)	40.0	20.0	307/216/166	1.4%/10.7%/21.5%
EP	0.707 (0.645-0.769)	23.8	14.2	294/-/395	2.7%/-/13.4%
EPclin	0.761 (0.707-0.815)	41.6	17.3	433/-/256	4.2%/-/16.9%

© 2018 Sestak et al. *JAMA Oncology.*

LR=Likelihood Ratio, CI=Confidence Interval, DR=Distant Recurrence, CTS=Clinical Treatment Score, IHC4=Immunohistochemical Score, BCI=Breast Cancer Index, RS=Recurrence Score, ROR=Risk of Recurrence Score, EP=EndoPredict, EPclin=EndoPredict clinical
Values are given for prognostic information of each signature on its own (LR-$\chi 2$), added prognostic value (ΔLR-$\chi 2$), number of women in each risk group, and 10-year distant recurrence (DR) risk for all women in years 0-10 (N=774) and years 5-10 (N=689).

© 2018 Sestak et al. *JAMA Oncology.*
